# Clinical and Molecular Diagnosis of Osteocraniostenosis in Fetuses and Newborns: Prenatal Ultrasound, Clinical, Radiological and Pathological Features

**DOI:** 10.3390/genes13020261

**Published:** 2022-01-28

**Authors:** Simonetta Rosato, Sheila Unger, Belinda Campos-Xavier, Stefano Giuseppe Caraffi, Laura Beltrami, Marzia Pollazzon, Ivan Ivanovski, Marco Castori, Maria Paola Bonasoni, Giuseppina Comitini, Peter G. J. Nikkels, Kristin Lindstrom, Christine Umandap, Andrea Superti-Furga, Livia Garavelli

**Affiliations:** 1 Medical Genetics Unit, Azienda USL-IRCCS di Reggio Emilia, 42123 Reggio Emilia, Italy; simonetta.rosato@ausl.re.it (S.R.); stefanogiuseppe.caraffi@ausl.re.it (S.G.C.); laura.beltrami.93@gmail.com (L.B.); marzia.pollazzon@ausl.re.it (M.P.); ivanovski@medgen.uzh.ch (I.I.); 2 Division of Genetic Medicine, Lausanne University Hospital (CHUV), CH-1011 Lausanne, Switzerland; sheila.unger@chuv.ch (S.U.); Belinda.Xavier@chuv.ch (B.C.-X.); asuperti@unil.ch (A.S.-F.); 3 Institut für Medizinische Genetik, Universität Zürich, 8902 Zürich, Switzerland; 4 Division of Medical Genetics, Fondazione IRCCS-Casa Sollievo della Sofferenza, 71013 San Giovanni Rotondo (FG), Italy; m.castori@operapadrepio.it; 5 Pathology Unit, Azienda USL-IRCCS di Reggio Emilia, 42123 Reggio Emilia, Italy; mariapaola.bonasoni@ausl.re.it; 6 Obstetrics and Gynecology Unit, Azienda USL-IRCCS di Reggio Emilia, 42123 Reggio Emilia, Italy; giuseppina.comitini@ausl.re.it; 7 Department of Pathology, University Medical Center Utrecht, 3584 CX Utrecht, The Netherlands; pnikkels@umcutrecht.nl; 8 Division of Genetics and Metabolism, Phoenix Children’s Hospital, Phoenix, AZ 85016, USA; Klindstrommd@gmail.com; 9 Medical Genetics, DMG Children’s Rehabilitative Services, Phoenix, AZ 85013, USA; Christine_Umandap@dmgaz.org

**Keywords:** osteocraniostenosis (OCS), Kenny-Caffey syndrome (KCS), *FAM111A*, cloverleaf skull, gracile bone dysplasia, hypoplastic spleen, asplenia, microphthalmia

## Abstract

Osteocraniostenosis (OCS, OMIM #602361) is a severe, usually lethal condition characterized by gracile bones with thin diaphyses, a cloverleaf-shaped skull and splenic hypo/aplasia. The condition is caused by heterozygous mutations in the *FAM111A* gene and is allelic to the non-lethal, dominant disorder Kenny-Caffey syndrome (KCS, OMIM #127000). Here we report two new cases of OCS, including one with a detailed pathological examination. We review the main diagnostic signs of OCS both before and after birth based on our observations and on the literature. We then review the current knowledge on the mutational spectrum of *FAM111A* associated with either OCS or KCS, including three novel variants, both from one of the OCS fetuses described here, and from further cases diagnosed at our centers. This report refines the previous knowledge on OCS and expands the mutational spectrum that results in either OCS or KCS.

## 1. Introduction

A lethal disorder characterized by thin, fragile long bones and craniofacial dysmorphism was initially reported by Kozlowski and Kan in 1988 and Maroteaux et al. in the same year [1,2]. The name “Osteocraniostenosis” (OCS) was first suggested in 1994 by Verloes et al. [3] after the finding of a consistent set of clinical and radiological anomalies in two fetuses and one newborn and a re-evaluation of three cases described by Kozlowski and Kan and by Maroteaux in 1988 [1,2]. These anomalies included extremely thin bones; brachydactyly; dysmorphic facial features such as a small philtrum, short nose, narrow mouth with tented upper lip vermilion, dysmorphic ears and ocular malformations (microphthalmia, aniridia); short and overmodeled tubular bones of the hands and feet; cloverleaf shape of the skull; and spleen aplasia or hypoplasia. Both males and females have been reported.

In 2006 Elliott et al. [4] disputed the term OCS for the disorder by stating that craniosynostosis is not a common finding, even if the skull shape radiographically resembles the trefoil or cloverleaf skull (“Kleeblattschädel”). This distinguishing skull configuration would rather be caused by severe hypomineralization of the cranial bones. The term “osteocraniosplenic” syndrome instead of OCS was suggested.

While patients affected by OCS are usually stillborn or die shortly after birth, the allelic disorder known as Kenny-Caffey syndrome (KCS) shows a milder phenotype with impaired skeletal development, small and dense bones, short stature and no life-threatening complications, except for recurrent hypocalcemia with low levels of parathyroid hormone (PTH), which requires continuous therapy with calcium and activated vitamin D [5,6,7]. The facial phenotype of KCS is characterized by a triangular face with frontal bossing, delayed closure of fontanels, small eyes with hypermetropia and dental anomalies. Other features are brittle long bones with narrow diaphysis and radiographically noticeable medullary stenosis.

Kenny and Linarelli were the first to report this condition in 1966 [8], while in 1967, Caffey [9] described the same family (affected mother and son) in more detail, pointing to cortical thickening with medullary stenosis of long bones, osteosclerosis of cranial bones and sparing of pelvic and vertebral bones. Another family with dominant transmission of the Kenny syndrome, from an affected mother to one daughter and two sons, was reported by Majewski et al. in 1981 [10].

Subsequently, a superficially similar disorder was described that includes hypoparathyroidism, short stature and developmental delay, and is inherited as a recessive rather than a dominant trait [11,12,13,14,15,16]. While first described as “recessive variant of the Kenny–Caffey syndrome”, when the gene responsible for this latter condition was isolated, it was named “Kenny-Caffey syndrome” (KCS); this is of course at odds with the original descriptions of the syndrome as a dominant trait. Currently, the OMIM catalogue perpetuates this error in listing “KCS type 1” (OMIM #244460) as recessive and linked to the *TBCE* gene, while the original, dominant KCS is listed as “KCS type 2” (OMIM #127000). In fact, the recessive KCS type 1 would better be called the Sanjad-Sakati syndrome, also known as hypoparathyroidism-retardation-dysmorphism syndrome (HRDS; OMIM #241410).

OCS and KCS share some common features: microphthalmia, a triangular face with frontal bossing, a spread of bone density associated with brittle and thin skeletal elements and hypocalcemia, which was not detected in Verloes’s original report [3] but later noticed in most of the OCS patients who survived beyond the perinatal period [7]. This evidence has led Unger et al. [7] to believe that OCS and KCS might be allelic disorders, and this hypothesis was confirmed by the finding that all cases they studied had monoallelic de novo variants in *FAM111A*, which is located on chromosome 11q12.1. A previously unknown role of *FAM111A* in the regulation of PTH has been speculated, which would cause hypoparathyroidism and compromise normal skeletal development.

It has been argued by several authors [4,17,18,19] that OCS is quite similar to Hallerman-Streiff syndrome (HSS). In fact, both of them show skull and long bone anomalies, but while HSS is not lethal, OCS often is; in addition, HSS has not been shown to have the characteristic facies of OCS or splenic hypoplasia.

Here we describe two clinical cases of fetuses with typical findings of OCS. Furthermore, we review the previous literature to define the most prominent prenatal ultrasound features, the main clinical and radiological aspects in the fetus and newborn and the most important features of the pathological examination.

## 2. Materials and Methods

All examinations and procedures described were conducted according to Good Clinical Practice and the Declaration of Helsinki (64th WMA General Assembly, Fortaleza, Brazil, October 2013). Data treatment was performed in accordance with Privacy Law (European regulation GDPR 2016/679). Radiological examinations, histopathological analysis and genetic testing were performed in the course of normal clinical practice and according to standard procedures, as described previously [7]. Signed informed consent was obtained from the subjects’ parents for genetic testing and for the publication of pictures and clinical data.

## 3. Patients and Results

### 3.1. Clinical Case #1

The first clinical case we considered was the fetus of a non-consanguineous couple (mother from Romania and father from Albania) whom we counseled during their first pregnancy. Prenatal ultrasound at 20 (+6) weeks of gestation found cloverleaf skull, microphthalmia, microretrognathia, thoracic hypoplasia, short long bones (<5th centile) and flexed feet. The karyotype on amniotic fluid was 46, XY.

**Figure 1:**
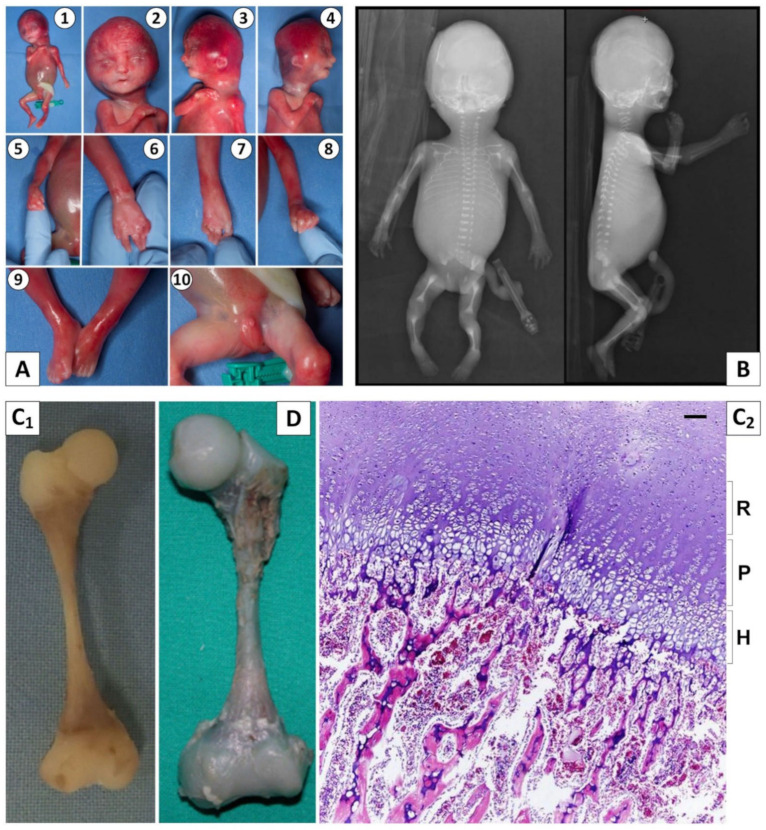
Clinical case #1. (**A1**–**4**) Frontal bossing, bitemporal narrowing with supra-auricular bulging and parietal bossing, short palpebral fissures, hypertelorism, narrow mouth, low-set ears. (**A5**–**8**) Camptodactyly of proximal interphalangeal joints of the 2nd–5th fingers. (**A9**) Bilateral short hallux. (**A10**) Micropenis. (**B**) Babygram X-rays: underossification of the skull, 11 pairs of thin ribs, mild platyspondyly, mild irregular cervical vertebrae, thin long bones with increased density, metaphyseal flaring and with obliteration of the medullary cavity. (**C**,**D**) Pathological examination of femur: (**C1**) macroscopically, the femur had a thin diaphysis with splayed metaphyses, and appeared similar to the picture (**D**) originally presented by Unger et al. [7]. (**C2**) Histological examination: architecture of the growth plate of the proximal femur showing a normal resting cartilage (R), a rarefied proliferating zone (P) and narrow cartilage lacunae in the hypertrophic zone (H). Scale bar = 25 μm.

Molecular analysis of the gene *FGFR3* on amniotic fluid—requested because the ultrasound findings of micromelia and cloverleaf skull had led to the hypothesis of thanatophoric dysplasia type II—resulted normal. After termination of the pregnancy at 22 weeks, the fetus showed a weight of 320 g (<3rd centile), a crown-heel length of 24.5 cm (<3rd centile), a head circumference of 17 cm (<3rd centile), frontal bossing, a narrow forehead with supra-auricular bulging and parietal bossing, a wide anterior fontanelle (3 by 4 cm), short palpebral fissures, hypertelorism, a narrow mouth, low-set ears, camptodactyly (contracture of the proximal interphalangeal joints of the 2nd–5th fingers), a bilateral short hallux, a ventricular septal defect (VSD), micropenis and asplenia. The main features of the objective examination of the fetus are shown in Figure 1A.

Babygram X-rays displayed decreased skull ossification, 11 pairs of thin ribs, mild platyspondyly, mild cervical vertebral dysplasia, slender long bones with flared metaphyses and increased density due to stenosis of the medullary cavity (Figure 1B).

Pathological examination of the femur revealed features similar to those reported in the literature [7], with grossly normal cartilaginous epiphysis but extremely thin diaphyses and broad metaphyses (Figure 1C). Histologic examination of the growth plate of the proximal femur showed a normal epiphyseal resting cartilage with a rarefied proliferating zone and hyperplastic chondrocytes with narrow cartilage lacunae in the hypertrophic zone.

The combination of clinical and radiographic features suggested the diagnosis of OCS. Sanger sequencing of the *FAM111A* gene on DNA from fetal and parental blood revealed that the fetus had a de novo heterozygous variant NM_001312909.2:c.1026_1028del, NP_001299838.1:p.(Ser343del), already associated with OCS [7].

### 3.2. Clinical Case #2

**Figure 2:**
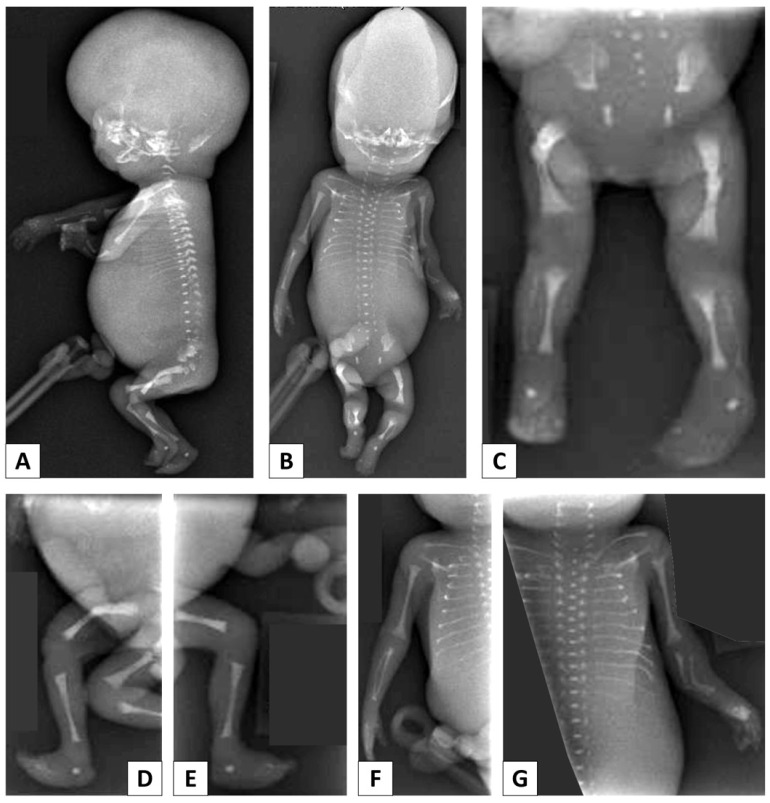
Clinical case #2: whole-body X-rays. (**A**–**E**) Reduced long bone length, thin long bones, bilateral femoral fractures with bowing. (**F**,**G**) Fractures with deformity of the left radius and ulna. (**A**,**B**,**F**,**G**) Gracile and irregular ribs, with fractures. (**C**,**F**,**G**) Bowing of the fibula and ulna. (**B**–**G**) Enlarged metaphyses of the tibia, femora and humeri.

The second case was the female fetus of a non-consanguineous couple. The pregnancy was terminated due to fetal anomalies. Labor was induced at the 21st (+2) week; the fetus was small for its gestational age, with a weight of 160 g (<3rd centile). The head was large; the cranial vault was undermineralized and most of its bones appeared to be absent (Figure 2A,B).

The lateral ventricles of the brain were enlarged. The fetus featured small rudimentary ears, a flattened wide nose and prominent lips. Small effusions of edematous tissues were found in the pleural cavities and abdomen, along with edematous placenta. The body displayed short limbs, echogenic bowel and a small spleen.

Whole-body X-rays (Figure 2) revealed bilateral femoral fractures with bowing, as well as fractures with deformity of the left radius and ulna. Long bone length was reduced and was closer to 16 weeks of gestation. The ribs were gracile and irregular in appearance. No definite callous formation was identified, but some of the irregularity may be due to fractures. The shafts of the long bones were thinned, with bowing of the fibula and ulna in particular. The metaphyses of the tibiae, femora and humeri appeared to be disproportionally enlarged. 

A pathology report detailing the microscopic analysis of the brain indicated a maturation consistent with the stated gestation of approximately 21 weeks, but noted striking necrosis and calcification of the basal ganglia and of the thalamic region.

As in the previous case, evidence led us to consider the diagnosis of OCS as the most likely. Molecular testing via Sanger sequencing revealed a novel missense variant in *FAM111A* exon 5: NM_001312909.2:c.1542G>T, NP_001299838.1:p.(Met514Ile). We were unable to test the parents, but the same variant, which is absent in the reference population database gnomAD v2.1.1 (Genome Aggregation Database, gnomad.broadinstitute.org/, accessed on June 2021), had already been found de novo in another fetus with OCS during a diagnostic routine at the Lausanne laboratory (not available for publication). Therefore, according to the recommendations of the American College of Medical Genetics [20], the variant was classified as likely pathogenic.

## 4. Discussion

Both fetuses reported here had some typical features already associated with several other cases of OCS syndrome: intrauterine growth restriction (IUGR), dysmorphism of the head, face and ears with frontal bossing, bitemporal narrowing with supra-auricular bulging and parietal bossing, an undermineralized cloverleaf-shaped skull, thin long bones with increased density, flared metaphyses and obliteration of the medullary cavity, and a hypoplastic or absent spleen. The first case also had micropenis and camptodactyly.

We reviewed the previous literature to further delineate the main prenatal ultrasound, clinical, radiological and pathological features of OCS. We considered both the cases who had a molecular confirmation and those without analysis of the *FAM111A* gene but with a compatible clinical picture. Among the latter, several are actually familial cases, with similarly affected siblings from healthy parents and/or with established consanguinity of the parents. Although germinal mosaicism cannot be ruled out, it seems an unlikely explanation given the number of these occurrences. It was recently proposed that the pathogenic mechanism correlating *FAM111A* defects with OCS involves PTH dysfunction or deficiency [7]. This may lead to the reasonable conjecture that defects in other genes involved in embryonic development and with a prominent role in PTH synthesis/regulation can engender a similar phenotype, possibly with an autosomal recessive mode of inheritance.

Table 1 lists the clinical characteristics reported in OCS and their occurrence out of the number of cases evaluated for the specific feature, counting separately for *FAM111A*-positive cases, all non-tested cases and non-tested cases without suspicion of recessive inheritance. The types of examination that may be employed to detect each feature are also indicated.

Based on the data from cases with *FAM111A* testing, fragile bones with defects in skull shape and ossification, along with growth delay, are the most distinctive characteristics of OCS. Defining the other notable features based on the clinical reports is not straightforward: some cases are newborns, while most are fetuses at different times of gestation, with some showing different degrees of maceration. Therefore, we choose to exclude a few non-tested cases which had insufficient data and did not have a definite brittle bone aspect.

Overall, whenever prenatal ultrasound was performed, the most prominent clinical features detected were a cloverleaf-shaped skull (11 out of 17 cases with at least one sonography reported), IUGR (15/17 cases), limb undergrowth (16/17 cases) and occasionally intrauterine bone fractures (7/17 cases) [3,4,22,23,24,25,26,28,29]. Most of these features were already apparent at 20 weeks of gestation (cfr. clinical case #1 in this report and [22,29]), but in at least one report growth had appeared normal up to 14 weeks [22]. In a few cases, a more thorough ultrasound examination was able to reveal additional diagnostic features, such as cranial vault hypomineralization, abnormal head circumference, flat facial profile, microphthalmia, acromicria, platyspondyly [4,23,29].

Head and face characteristics were quite consistent, with most fetuses and newborns showing a combination of frontal bossing, supra-auricular bulging, a flat face, short nose and low-set ears. Microphthalmia is a less common clinical feature; however, it can be helpful to suspect the diagnosis.

Micropenis in males was a consistent finding among the few cases with molecular confirmation of a *FAM11A* variant [7], including our clinical case #1. In previous reports it had been clearly documented in only a single case [4]; however, data were only available for less than half of the male fetuses/newborns considered. This suggests that micropenis might be a significant but underreported feature of OCS.

The radiological evidence was the most evocative aspect of OCS. In addition to an often-dysmorphic appearance, the skull usually showed markedly delayed ossification, frequently with missing bones and/or a wide anterior fontanelle. In nearly all reported cases, the long bones had a distinctive aspect with diaphyseal stenosis and metaphyseal flaring. All but one reports indicated thin ribs, and platyspondyly was observed in at least two-thirds of the cases. Bone fractures at birth, a consequence of these ossification defects, occurred less frequently but can be very useful for the diagnostic process.

Pathological examination can reveal important anatomical and histological aspects. Splenic hypoplasia or aplasia was the most common finding after the skeletal and craniofacial features and it may be useful to suspect this condition. Defects in other organs were present but less frequent, and included lung hypoplasia, hepatomegaly or other liver dysfunctions and congenital heart disease. Thymic hypoplasia was also present but had only been looked for in a small number of cases, and further investigations are required to establish its frequency. Similarly, extramedullary hemopoiesis might also be a poorly looked-for but consistent feature of OCS, since it was observed in 2 out of 6 cases with confirmed *FAM111A* mutation and 2 out of 4 cases with no molecular testing.

Along with the features revealed by prenatal ultrasound and babygram X-rays, the histological examination of bone growth plates is emerging as a very important criterion to support the diagnosis of OCS, as Spear et al. reviewed [31]. Several of the examined cases showed varying degrees of growth plate irregularity, generally involving short/disorganized columns of pleomorphic chondrocytes in the proliferating and/or hypertrophic zone. Macroscopic and histological examination of a femur from our clinical case #1 was well within the spectrum of abnormalities described so far. In fact, it showed marked similarities with case #7 from Unger et al. [7]: both displayed a thin diaphysis and grossly enlarged metaphyses, and the histological aspect of the growth plate was mildly disorganized (Figure 1C,D).

KCS patients, recently reviewed by Cheng et al., have a milder phenotype overall [32]. They share elements of defective mineralization with OCS, but at the milder end of the spectrum, with features such as delayed closure of the anterior fontanelle rather than severely decreased skull ossification [7,32]. KCS patients are characterized by hypoparathyroidism and hypocalcemia; low calcium levels have also been detected in OCS cases, supporting the hypothesis of a PTH dysfunction underlying both conditions [7].

**Figure 3:**
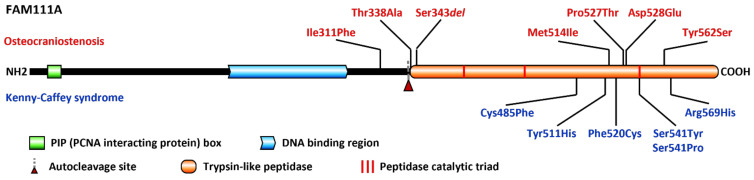
Scheme of the FAM111A protein and known pathogenic variants associated with either osteocraniostenosis (OCS, **top**) or Kenny-Caffey syndrome (KCS, **bottom**). Cys485Phe was reported in [33], Ser541Tyr/Pro were reported in [5,32], respectively, Tyr562Ser was reported in [21], while all other variants were observed at the Laboratory of Genetics at CHUV ([7] and this report). The domain in orange has homology to trypsin-like peptidases, including an untested catalytic triad purportedly composed of Ser541/His385/Asp439 (red bars).

Figure 3 lists the OCS- and KCS-associated *FAM111A* variants reported in the literature, and novel variants observed during diagnostic activity at the Lausanne Genetics Laboratory. These include the Met514Ile variant also described here in clinical case #2, as well as the de novo variants Phe520Cys, seen in a child with KCS not reported before, and Phe311Ile, identified in an OCS case with IUGR, macrocephaly and facial dysmorphisms (ClinVar SCV001439356.1). Most variants are clustered together near the protein C-terminus, and a definite genotype–phenotype correlation, explaining the different severity of OCS and KCS, has not been established yet.

Through homology comparison and functional studies, the protein encoded by *FAM111A* is emerging as a serine protease putatively involved in DNA replication and response to replication stress [34,35,36,37,38]. FAM111A colocalizes with the replication fork, possibly interacting with single-strand DNA through its central region, while its C-terminal portion features a conserved Ser/His/Asp catalytic triad and acts as a chymotrypsin-like peptidase (Figure 3). Although its exact biological targets are still being investigated, auto-cleavage activity has been documented, both in cis and in trans, suggesting that multiple copies of FAM111A might interact with other factors to form active complexes [36,37,38]. Interestingly, the SWISS–MODEL server reports a high homology with serine protease HtrA2, which is known to function as a trimer [39].

Haploinsufficiency is unlikely to explain the pathogenetic mechanism. As already noted [7], null alleles are present in reference population databases, including the latest releases of gnomAD (v2.1.1, v3.1.2, accessed June 2021), and have not been associated with known cases of OCS or KCS. In vitro experiments on *FAM111A* knockout cell lines indicate a negligible effect on cell viability, except for an increased sensitivity to specific agents inducing DNA replication stress [36,37]. Future studies on model organisms (currently in planning, according to the International Mouse Phenotyping Consortium) are needed to support these observations.

All OCS- or KCS-associated variants identified to date lead to single amino acid substitutions or deletions. Based on 3D models predicting that most variants occupy a limited area on the protein surface in the vicinity of the protease domain, a dominant gain-of-function pathogenetic mechanism was proposed [7]. Accordingly, recent experimental studies suggested that known disease-associated variants increase an intra- and intermolecular autocleavage mechanism and display protease activity-dependent cytotoxicity [37,38]. It is tempting to speculate that OCS-associated variants occurring near the cleavage site might influence autoproteolytic specificity (e.g., Ser342), while variants clustered in the C-terminal peptidase-homology domain might affect its catalytic activity [37]. Despite these advances, functional evidence on FAM111A is still limited, in particular concerning its targets and the subcellular localization of its variants, and has not yet provided an explanation for the gap in severity between OCS and KCS. Homology- and prediction-based structural models, such as those generated by AlphaFold (accessed July 2021) [40], show high confidence only in a restricted area that does not encompass all reported variants, and the putative catalytic triad has not been directly tested yet [38]. Further investigations will be needed, as well as the description of a larger number of cases and their associated variants, in order to define the pathogenetic mechanism of these allelic conditions.

## 5. Conclusions

OCS can be recognized prenatally in the presence of evocative elements such as craniostenosis or cloverleaf skull; thin and brittle bones; asplenia, usually co-occurring with the nonspecific findings of IUGR; and limb undergrowth.

Molecular confirmation of the diagnosis is essential for the parents as it allows geneticists and other healthcare professionals to establish a low recurrence risk, and to plan or limit prenatal investigations accordingly.

## Figures and Tables

**Table 1:** Skeletal and clinical features of osteocraniostenosis (OCS).

	Prenatal Ultrasound	Babygram X-rays	Objective Exam	Anatomopathology ^a^	OCS Cases, *FAM111A* Mutation Detected									OCS Cases, No *FAM111A* Analysis	
					Case #1	Case #2							Test+, Total ^b,c^	Test−, Non-Recessive ^b,d,e^	Test−, Total ^b,d^
**References**					This study	This study	[7]					[21]		[1,2,3,4,22,23,24,25,26,27,28,29,30]	
**Case # (# in original reference)**					1	2	3 (6)	4 (7)	5 (8)	6 (9)	7 (10)	8	8/8	18/30	30/30
***FAM111A* variant (hg19)** **NM_001312909 NP_001299838**					c.1026_1028del p.S343del	c.1542G > T p.M514I	c.1026_1028del p.S343del	c.1026_1028del p.S343del	c.1012A > G p.T338A	c.1583A > G p.A528G	c.1579C > A p.P527T	c.1685A > C p.Y562S			
**Sex**					M	F	M	M	M	M	M	F	6M 2F	8M 7F	13M 14F
**Age at follow-up**					22 w (TOP)	21 w (TOP)	3 d (†)	25 d (†)	2 m (†)	20 m	8 m (†)	20 w (TOP)			
**Anthropometric features**															
Intrauterine growth restriction	U	X	O		+	+	+	+	+	+	+	+	**8/8**	**14/16**	**20/27**
Microcephaly	U	X	O		+	+	NA	NA	NA	NA	NA	NA	2/2	**7/9**	**12/21**
Macrocephaly	U	X	O		−	−	NA	NA	NA	NA	NA	NA	0/2	**1/10**	**4/21**
**Craniofacial features**															
Cloverleaf-shaped skull	U	X	O		+	NA	+	+	+	+	+	+	**7/7**	**10/14**	**16/26**
Large anterior fontanelle		X	O		+	NA	NA	NA	NA	NA	NA	+	2/2	**10/11**	**14/20**
Frontal bossing	U	X	O		+	NA	NA	+	NA	NA	NA	+	3/3	**10/12**	**20/24**
Supra-auricular bulging/parietal bossing	U	X	O		+	NA	NA	+	NA	NA	NA	NA	2/2	**10/11**	**15/18**
Mid-face hypoplasia	U		O		+	NA	NA	NA	NA	NA	NA	NA		**10/12**	**15/22**
Flat face	U		O		+	NA	NA	NA	NA	NA	NA	NA		**13/13**	**18/23**
Hypertelorism	U		O		+	NA	NA	NA	NA	NA	NA	+	2/2	**3/13**	**11/25**
Hypotelorism	U		O		−	NA	NA	NA	NA	NA	NA	−	0/2	**5/13**	**5/24**
Small palpebral fissures	U ^f^		O		+	NA	NA	NA	NA	NA	NA	NA		**8/10**	**8/19**
Microphthalmia	U ^e^		O		+	NA	−	+	+	−	−	−	**3/7**	3/6	**4/16**
Depressed nasal bridge	U		O		+	NA	NA	NA	NA	NA	NA	+	2/2	**6/10**	**10/19**
Short nose	U		O		+	+	NA	NA	NA	NA	NA	+	3/3	**13/13**	**20/23**
Narrow mouth	U ^f^		O		+	NA	NA	NA	NA	NA	NA	−	1/2	**10/12**	**12/21** ^g^
Microretrognathia	U		O		+	NA	NA	NA	NA	NA	NA	+	2/2	0/4	**8/16**
Low-set ears	U		O		+	NA	NA	+	NA	NA	NA	+	**3/7**	**11/12**	**17/21**
Short neck	U		O		−	NA	NA	NA	NA	NA	NA	NA		**6/11**	**10/20**
**Skeletal features**															
Decreased skull ossification	U	X		A	+	+	+	+	+	+	+	+	**8/8**	**14/15**	**23/25**
Thoracic hypoplasia	U	X	O		+	NA	NA	NA	NA	NA	NA	−	1/2	**6/12**	**12/23**
Thin ribs	U ^f^	X		A	+	+	NA	+	NA	NA	NA	−	**3/4**	**18/18**	**30/30**
11 pairs of ribs		X		A	+	NA	NA	NA	NA	NA	NA	−	1/2	3/8	**3/14**
Slender long bones		X		A	+	+	+	+	+	+	+	+	**8/8**	**18/18**	**30/30**
Flared metaphyses		X		A	+	+	+	+	+	+	+	+	**8/8**	**17/18**	**28/29**
Stenosis of the medullary cavity of the long bones		X		A	+	NA	+	+	+	+	+	NA	**6/6**	**14/14**	**22/22**
Limb undergrowth	U	X	O		+	+	NA	NA	NA	NA	NA	+	3/3	**14/16**	**20/28**
Acromicria	U	X	O		+	NA	NA	NA	NA	NA	NA	NA		**9/9**	**9/13**
Brachydactyly	U ^f^	X	O		NA	NA	NA	NA	NA	NA	NA	NA		**11/11**	**16/21**
Platyspondyly	U ^f^	X		A	+	NA	NA	NA	NA	NA	NA	NA		**9/11**	**15/20**
Bone fractures	U	X			−	+	−	−	+	−	−	−	2/8	**13/18**	**17/29** ^h^
**Other clinical features**															
Histological anomalies in the growth plate of the long bones ^i^				A	+	NA	NA	+	NA	NA	NA	NA	2/2	**7/9**	**13/15**
Aplasia/hypoplasia of the spleen				A	+	+	+	−	NA	NA	NA	+	**4/5**	**11/13**	**14/19**
Pulmonary hypoplasia	U			A	−	NA	NA	NA	NA	NA	NA	+	1/2	**4/11**	**7/19**
Hepatomegaly	U			A	−	NA	NA	NA	NA	NA	NA	NA	0/1	3/6	**5/13**
Extramedullary hematopoiesis				A	+	NA	NA	+	NA	NA	NA	NA	2/2	1/2	2/4
Congenital heart defects	U			A	−	NA	NA	NA	NA	NA	NA	−	0/2	2/4	**3/12**
Brain abnormalities	U ^j^			A	−	+	NA	NA	NA	NA	NA	+	2/3	3/6	**4/11**
Hydrocephalus	U		O		−	NA	−	−	−	+	+	−	**2/7**	**0/9**	**2/19**
Hydrops fetalis	U		O		−	NA	NA	NA	NA	NA	NA	+	1/2	**1/10**	**2/18**
Hypoplasia of the nails			O		NA	NA	NA	NA	NA	NA	NA	NA		8/9	**8/14**
Foot anomalies	U		O		+	NA	NA	NA	NA	NA	NA	NA		5/7	**8/13**
Micropenis	U		O		+	NA	+	+	−	+	+	/	**5/6**	1/4	1/6
Hypocalcemia/Hypoparathyroidism					NA	NA	+	+	+	+	+	NA	**5/5**	0/0	0/1

Abbreviations: #, number; Test+/−, tested or non-tested for *FAM111A*; TOP, termination of pregnancy; M, male; F, female; d, days; w, weeks; m, months; NA, not available/not known. Notes: ^a^ excluding features already evident at clinical observation; ^b^ number of cases presenting with the listed feature out of the number of cases formally evaluated for the feature; ^c^ presented in bold when at least 50% of the cases were evaluated; ^d^ presented in bold when at least 9 cases were evaluated; ^e^ i.e., excluding siblings and other cases with suspected recessive inheritance; ^f^ detection possible, but not always optimal; ^g^ usually with inverted U/V-shaped mouth; ^h^ at birth; at least 8 known to be intrauterine; ^i^ irregular shape/distribution of chondrocytes; ^j^ depending on the type of brain anomaly; † deceased at given age.

## Data Availability

All data is available upon reasonable request by contacting the corresponding author.
